# The association between the consumption of raw *Kudoa septempunctata*–infected farmed *Paralichthys olivaceus* and gastrointestinal symptoms

**DOI:** 10.4178/epih.e2026003

**Published:** 2026-01-19

**Authors:** Jihye An, En-Joo Jung, Soon-Ok Lee, Jong-Hoon Choi, JungHee Kim, Sung-Jong Hong, Sung-Hee Hong, Jung-Won Ju, Hyungjun Kim, Kwang-Pil Ko

**Affiliations:** 1 Department of Epidemic Intelligence Service, Incheon Communicable Diseases Center, Incheon, Korea; 2 Department of Public Healthcare Center, Seoul National University Hospital, Seoul, Korea; 3 Department of Preventive Medicine, Seoul National University College of Medicine, Seoul, Korea; 4 Department of Medical Research Center for Bioreaction to Reactive Oxygen Species, Biomedical Science Institute, School of Medicine, Graduate School, Kyung Hee University, Seoul, Korea; 5 Division of Vectors and Parasitic Diseases, Korea Disease Control and Prevention Agency, Cheongju, Korea; 6 Incheon Metropolitan City Institute of Public Health and Environment, Incheon, Korea; 7 Chung-Ang University, Seoul, Korea; 8 Division of Infectious Disease Control, Korea Disease Control and Prevention Agency, Cheongju, Korea; 9 Clinical Preventive Medicine Center, Seoul National University Bundang Hospital, Seongnam, Korea

**Keywords:** Cohort studies, Disease outbreaks, Foodborne diseases, Gastrointestinal diseases, Kudoa septempunctata, Risk factors

## Abstract

**OBJECTIVES:**

*Kudoa septempunctata* has been identified as the causative agent of food poisoning following the consumption of raw farmed *Paralichthys olivaceus*. However, cohort studies providing robust evidence for an association between *K. septempunctata* and gastrointestinal symptoms remain limited. This prospective cohort study investigated the association between the consumption of *K. septempunctata*–infected farmed *P. olivaceus* and the occurrence of gastrointestinal symptoms.

**METHODS:**

Individuals who purchased raw farmed *P. olivaceus* between 2020 and 2021 were selected as the study population. Study data included 2 rounds of questionnaire surveys administered before and after consumption, 2 muscle specimens obtained from each purchased fish, and human biological specimens collected from individuals who developed gastrointestinal symptoms within 24 hours after consumption. Data were analyzed using the chi-square test and t-test, and the association between consumption of *K. septempunctata*–infected farmed *P. olivaceus* and gastrointestinal symptoms was evaluated using relative risk estimates between exposure groups.

**RESULTS:**

The relative risk of gastrointestinal symptoms associated with exposure to *K. septempunctata*–infected *P. olivaceus* ranged from 71.2 (95% confidence interval [CI], 27.0 to 178.6) to 124.5 (95% CI, 43.5 to 355.0) across the 2 case definitions. A strong and statistically significant association was observed between exposure to *K. septempunctata*–infected *P. olivaceus* and the development of acute gastrointestinal symptoms.

**CONCLUSIONS:**

These findings indicate both an association and a causal relationship between consumption of *K. septempunctata*–infected farmed *P. olivaceus* and the onset of gastrointestinal symptoms.

## GRAPHICAL ABSTRACT

[Fig f2-epih-48-e2026003]

## Key Message

This study provides prospective evidence that consumption of raw halibut infected with *Kudoa septempunctata* is associated with gastrointestinal symptoms, with the severity of symptoms increasing with the intensity of infection and the amount consumed. These results address a gap in epidemiological knowledge and highlight the need for strengthened surveillance systems and prevention-focused policies.

## INTRODUCTION

*Kudoa septempunctata* was first reported during a mass food poisoning incident in Japan in 2010 and was subsequently identified as the causative pathogen responsible for symptoms following consumption of raw farmed *Paralichthys olivaceus* [1-4]. In Korea, exposure to *K. septempunctata* is relatively common because *P. olivaceus* is widely consumed [5,6]. Nevertheless, whether *K. septempunctata* definitively causes food poisoning symptoms remains a matter of debate [2,4,7-10].

*P. olivaceus* is the most commonly consumed marine species within the order *Pleuronectiformes* and accounted for 51% of all farmed marine fish production in 2013 [6,11]. As a result, several Korean studies have investigated the prevalence of *K. septempunctata* infection in *P. olivaceus* farms and have conducted related case-control studies [12].

Most existing studies are case reports or case-control investigations involving patients who developed gastrointestinal symptoms and, as such, cannot adequately substantiate a causal association between *K. septempunctata* and gastrointestinal symptoms [8,12,13]. Importantly, in many studies, consumed food specimens were unavailable, which precluded confirmation of infection in the raw fish [5,14,15]. In addition, biological studies have yielded inconsistent results due to variability in study populations and experimental methods, highlighting the need for epidemiological studies capable of more definitively clarifying this association [1,2,8,9,16-19].

The present study aimed to investigate the association between consumption of *K. septempunctata*–infected *P. olivaceus* (referred to in this manuscript as positive olive flounder) and the onset of gastrointestinal symptoms, as well as to evaluate the pathogenicity of *K. septempunctata*. The specific objectives were: (1) to analyze the prevalence of *K. septempunctata* infection among raw farmed *P. olivaceus*; and (2) to analyze the causal relationship between consumption of raw positive olive flounder and the onset of gastrointestinal symptoms.

## MATERIALS AND METHODS

### Study design

**Figure 1.**
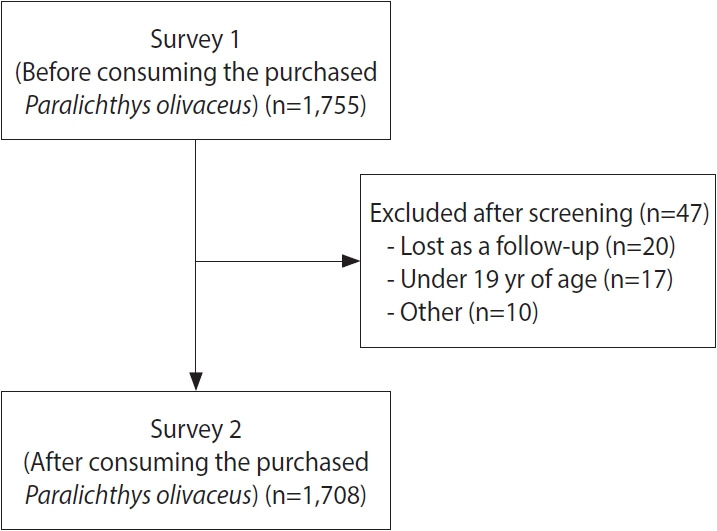
Flow chart of the cohort study design.

This prospective cohort study examined the incidence of gastrointestinal symptoms among individuals who purchased and consumed farmed *P. olivaceus* between 2020 and 2021 and investigated the association between gastrointestinal symptoms and *K. septempunctata* (Figure 1). Among the 1,755 individuals initially enrolled, participants who did not complete the second questionnaire, individuals younger than 19 years of age, and others (including participants who may have consumed multiple types of fish or who provided incorrect contact information) were excluded. As a result, a total of 1,708 participants were included in the final analysis.

To minimize potential bias, several measures were implemented: (1) researchers and investigators received standardized training to ensure consistency in study procedures and survey administration; (2) samples of raw fish were collected and tested for *K. septempunctata* infection before consumption by study participants; (3) participants were required to complete a second questionnaire within 24 hours after consuming raw fish, regardless of *K. septempunctata* infection status (Supplementary Material 1); and (4) for participants who developed symptoms, human biological specimens were examined to exclude potential causes other than *K. septempunctata* (Supplementary Material 2).

### Study participants and sample size

Individuals who purchased farmed *P. olivaceus* at seafood markets or seafood sections of retail markets in Seoul, Gyeonggi, and Incheon between 2020 and 2021 and who provided informed consent were enrolled in this study. The authors or trained surveyors explained the purpose of the study to individuals purchasing farmed *P. olivaceus*. Participants completed a pre-consumption survey (Survey 1) and provided a specimen of the *P. olivaceus* they had purchased. In addition, individuals who consumed farmed *P. olivaceus* purchased together were asked to complete a post-consumption survey (Survey 2) if they agreed to participate.

For sample size estimation, we assumed a type I error of 0.05 and a type II error of 0.2. Based on previous studies, the prevalence of *P. olivaceus* infected with *K. septempunctata* has been reported to range from 3% to 5%; therefore, we conservatively estimated a prevalence of 2% [3,10]. We further assumed a 50% attack rate and a 10% incidence of gastrointestinal symptoms among individuals consuming infected *P. olivaceus*. Under these assumptions, a total sample size of 735 participants was required.

### Identification of cases

Cases were defined based on the severity of gastrointestinal symptoms that developed within 24 hours after consumption of raw farmed *P. olivaceus*, as follows: (1) Case definition 1: Individuals who experienced at least 1 gastrointestinal symptom, such as diarrhea, vomiting, nausea, or stomach pain; (2) Case definition 2: Individuals who had 1 episode of diarrhea or vomiting along with other gastrointestinal symptoms, such as nausea or stomach pain.

### Collection of raw flounder specimens and examination for *K. septempunctata* infection

The Kudoa Rapido A rapid diagnostic test kit was used to detect Kudoa antibodies in raw *P. olivaceus*. In addition, to exclude false-negative results, approximately 10 g of tissue was collected from 2 separate sites in each fish, and *K. septempunctata* infection was confirmed through visual inspection. No other infectious agents were investigated.

### Human specimen testing from symptomatic individuals

More than 5 g of human specimens (vomitus or feces) were collected from participants who developed gastrointestinal symptoms after consuming fish infected with *K. septempunctata*. Specimens were stored at 4°C and examined using standard laboratory procedures, including protozoan analysis. Detailed information on test items for human specimens is provided in Supplementary Material 2.

### Statistical analysis

All statistical analyses were conducted using SAS version 9.4 (SAS Institute Inc., Cary, NC, USA), and statistical significance was defined as p-value<0.05. Data distributions and mean values between groups exposed and not exposed to *K. septempunctata* were compared using chi-square tests for categorical variables and independent *t*-tests for continuous variables. Relative risks (RRs) and corresponding 95% confidence intervals (CIs) are reported.

### Ethics statement

The study protocol was approved by the Institutional Review Boards of Gacheon University Gil Medical Center (GAIRB-2020-158) and Seoul National University Bundang Hospital (B-2107-696-301).

## RESULTS

### General characteristics

Participants were divided into 2 groups based on whether they consumed raw fish infected with *K. septempunctata*. The general characteristics of the study participants are summarized in Table 1. A total of 1,708 participants were included in the analysis, of whom 27 (1.6%) were exposed to *K. septempunctata* and 1,681 (98.4%) were classified as unexposed.

Among the participants, 754 (44.1%) were men and 954 (55.9%) were women. The most common age group among individuals who consumed raw fish was 50–59 years (n=458, 26.8%), followed by those aged ≥60 years (n=373, 21.8%) and those aged 40–49 years (n=314, 18.4%).

Methods of storing raw flounder before ingestion differed between groups. In the exposure group, storage methods included immediate ingestion (n=9, 33.3%), storage at room temperature (n=3, 11.1%), and refrigeration (n=15, 55.6%). In the non-exposure group, storage methods included immediate ingestion (n=174, 10.4%), room temperature storage (n=110, 6.5%), refrigeration (n=1,370, 81.5%), and freezing (n=27, 1.6%). The time from purchase of raw farmed *P. olivaceus* to consumption was within 1 hour (n=7, 25.9%), 1–2 hours (n=16, 59.3%), and 2–3 hours (n=4, 14.8%) in the exposure group, and within 1 hour (n=494, 29.4%), 1–2 hours (n=371, 22.1%), 2–3 hours (n=243, 14.5%), and ≥3 hours (n=573, 34.1%) in the non-exposure group.

The number of raw slices of farmed *P. olivaceus* consumed in the exposure group was 3–7 (n=6, 22.2%), 8–12 (n=6, 22.2%), 13–17 (n=6, 22.2%), 18–22 (n=4, 14.8%), and ≥23 slices (n=5, 18.5%). In the non-exposure group, the corresponding consumption categories were 3–7 (n=369, 22.0%), 8–12 (n=632, 37.6%), 13–17 (n=390, 23.2%), 18–22 (n=185, 11.0%), and ≥23 slices (n=105, 6.3%).

In the exposure group, 2 participants (7.4%) reported having food allergies, while 25 (92.6%) reported no food allergies. In the non-exposure group, 21 participants (1.3%) reported food allergies, 1,605 (95.5%) reported no food allergies, and 55 (3.3%) reported that they did not know their allergy status.

### Impact of *K. septempunctata*-infected *P. olivaceus* on risk

Table 2 presents the RRs for gastrointestinal symptoms in the exposure group according to case definition. Due to the small number of events, exact RRs and 95% CIs were calculated using the Koopman asymptotic score method for 2×2 contingency tables. For case definition 1, 8 participants in the exposure group and 7 participants in the non-exposure group developed symptoms, yielding an RR of 71.2 (95% CI, 27.0 to 178.6) for gastrointestinal symptoms in the exposure group compared with the non-exposure group. For case definition 2, 6 participants in the exposure group and 3 participants in the non-exposure group developed symptoms, yielding an RR of 124.5 (95% CI, 43.5 to 355.0) for gastrointestinal symptoms in the exposure group compared with the non-exposure group.

### Human specimen test results for symptomatic individuals

As shown in Supplementary Material 3, gastrointestinal symptoms such as diarrhea and nausea occurred more frequently and with greater severity among individuals who consumed larger amounts of raw positive olive flounder, particularly when the infection intensity exceeded the minimum infectious dose threshold reported in previous studies [4,12].

Standard testing of human specimens, including protozoan assays, yielded negative results for all tested agents except *K. septempunctata* [8,16-19] (Supplementary Material 4).

## DISCUSSION

Various *myxosporean* infections have been reported in farmed *P. olivaceus* in Korea, raising concerns regarding their persistence and potential impacts on aquaculture production [3,6,11]. *K. septempunctata* is a muscle parasite of *P. olivaceus*; however, it is not readily detected by microscopic examination during the early stages of infection [3,4,12,15,20,21].

In Japan, an epidemiological investigation of a mass food poisoning outbreak affecting 113 individuals in Ehime Prefecture and 8 other prefectures in October 2010 examined 60 specimens of *P. olivaceus* (farmed *P. olivaceus* in Korea and Japan) and identified a new species belonging to the order *Multivalvulid*, later named *K. septempunctata*, in the muscle tissue of farmed *P. olivaceus* imported from Korea. This strain was classified as the ST3 genotype, which differs from the *K. septempunctata* strains found in *P. olivaceus* inhabiting waters surrounding Japan (ST1 and ST2) [1,8,16]. The Japanese Ministry of Health, Labor, and Welfare subsequently recognized this protozoan species as a cause of food poisoning and designated the condition as Kudoa food poisoning [2-4,22].

To date, the association between *K. septempunctata* and gastrointestinal symptoms has remained unclear [5,7-9,14-17,19]. The present prospective cohort study aimed to analyze the prevalence of *K. septempunctata* infection in the raw flesh of farmed *P. olivaceus* and to evaluate the causal relationship between consumption of raw positive olive flounder and the onset of gastrointestinal symptoms. The exposure group exhibited a higher RR of gastrointestinal symptoms than the non-exposure group, and analyses stratified by case definition demonstrated that consumption of raw positive olive flounder was associated with the occurrence of gastrointestinal symptoms. Notably, higher infection intensity in positive olive flounder and greater amounts consumed were associated with more severe gastrointestinal symptoms. Among participants who consumed *P. olivaceus* infected with *K. septempunctata*, symptom presence, frequency, and incubation period are summarized in Supplementary Material 3. The raw positive olive flounder sample numbers presented in Supplementary Materials 3 and 4, as well as the exposure numbers of symptomatic participants according to raw positive olive flounder sample number, were arbitrarily assigned by the researchers. In a previous study, the minimum ingestion threshold for the development of gastrointestinal symptoms was reported as 7.2×10⁷ spores/g, and in the present study, gastrointestinal symptoms were observed in exposed participants as the infection intensity of raw positive olive flounder approached this minimum intake threshold [4,12]. A previous study also reported relatively consistent findings regarding the incubation period and clinical manifestations of gastrointestinal symptoms following consumption of raw positive olive flounder [2,4].

Some studies have argued that the association between *K. septempunctata* and gastrointestinal symptoms cannot be conclusively established because other bacterial pathogens with similar epidemiological characteristics, such as *Staphylococcus aureus* and *Bacillus cereus*, may also be involved [8,15,19].

Although human studies investigating the biological mechanisms underlying gastrointestinal symptoms remain limited, several studies have suggested that *K. septempunctata* spores ingested through consumption of infected marine fish infiltrate intestinal epithelial cells and induce gastrointestinal symptoms [2,4,18,23]. In addition, toxicological studies have demonstrated that ingestion of 1.0×10⁷ spores/g of *P. olivaceus* induced vomiting in *Suncus murinus* [4].

We conducted a prospective epidemiological study to support previous findings regarding the onset of gastrointestinal symptoms following consumption of raw flesh from *K. septempunctata*–infected fish [2,4,7-10]. A portion of the raw fish consumed by participants was collected and tested for *K. septempunctata* infection. Through this approach, we identified the consumption histories of participants who consumed raw fish infected with *K. septempunctata*, as well as the presence or absence, frequency, and intensity of gastrointestinal symptoms. Our findings indicate that both the frequency and severity of gastrointestinal symptoms tended to be greater among participants who consumed larger amounts of raw fish with higher levels of *K. septempunctata* infection, as detailed in Table 2 and Supplementary Material 3. The primary significance of this study lies in demonstrating an association between consumption of raw positive olive flounder and gastrointestinal symptoms. Nevertheless, several limitations should be considered. First, farmed *P. olivaceus* samples were periodically tested for *K. septempunctata* at an external laboratory, which occasionally delayed the collection of human biological specimens, even when fish samples tested positive. As a result, evidence regarding the onset of gastrointestinal symptoms in relation to infection intensity may be limited. Second, Survey 2 was administered within 24 hours after consumption to assess symptom frequency, which may have introduced recall bias. Additional limitations include reliance on subjective reporting of symptom frequency, except for diarrhea and vomiting, and the use of symptom frequency alone as a proxy for symptom severity. Third, only 2 cases of gastrointestinal symptoms occurred following consumption of raw positive olive flounder. However, given that many participants exhibited gastrointestinal symptoms with clinical manifestations and incubation periods consistent with *K. septempunctata*–associated illness, and that no other causative pathogens were identified in human biological specimens, it is reasonable to infer that *K. septempunctata* was the causative agent in these cases. To address these limitations, further studies involving broader geographic regions and larger populations are warranted.

Despite these limitations, our findings demonstrate an association between consumption of raw positive olive flounder and the onset of gastrointestinal symptoms. These results underscore the need for policies and guidelines aimed at preventing gastrointestinal illness associated with consumption of raw infected fish.

Our findings further suggest an epidemiological association between ingestion of raw *K. septempunctata*–infected flounder and the occurrence of infectious gastrointestinal disease. Accordingly, policies to strengthen early management and continuous monitoring of raw farmed *P. olivaceus* prior to distribution are needed to prevent and reduce gastrointestinal symptoms caused by *K. septempunctata* infection.

## Figures and Tables

**Figure f2-epih-48-e2026003:**
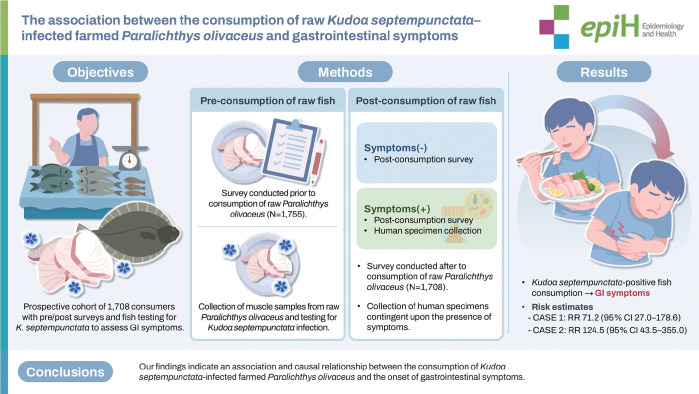


**Table 1.** General characteristics of the study population

Characteristics	Total	Exposure	Non-exposure	p-value
Total	1,708 (100)	27 (1.6)	1,681 (98.4)	
Gender				0.453
Men	754 (44.1)	10 (37.0)	744 (44.3)	
Women	954 (55.9)	17 (63.0)	937 (55.7)	
Age (yr)				0.235
≤29	302 (17.7)	7 (25.9)	295 (17.6)	
30–39	261 (15.3)	5 (18.5)	256 (15.2)	
40–49	314 (18.4)	2 (7.4)	312 (18.6)	
50–59	458 (26.8)	10 (37.0)	448 (26.7)	
≥60	373 (21.8)	3 (11.1)	370 (22.0)	
Storage method				0.001
Immediate intake	183 (10.7)	9 (33.3)	174 (10.4)	
Room temperature	113 (6.6)	3 (11.1)	110 (6.5)	
Refrigerated	1,385 (81.1)	15 (55.6)	1,370 (81.5)	
Frozen	27 (1.6)	0 (0)	27 (1.6)	
Time from purchase to intake (hr)				<0.001
<1	501 (29.3)	7 (25.9)	494 (29.4)	
1–2	387 (22.7)	16 (59.3)	371 (22.1)	
2–3	247 (14.5)	4 (14.8)	243 (14.5)	
>3	573 (33.5)	0 (0)	573 (34.1)	
Amount consumed (pieces)				0.083
3–7	375 (22.0)	6 (22.2)	369 (22.0)	
8–12	638 (37.4)	6 (22.2)	632 (37.6)	
13–17	396 (23.2)	6 (22.2)	390 (23.2)	
18–22	189 (11.1)	4 (14.8)	185 (11.0)	
≥23	110 (6.4)	5 (18.5)	105 (6.3)	
Allergy history				0.015
Yes	23 (1.3)	2 (7.4)	21 (1.3)	
No	1,630 (95.4)	25 (92.6)	1,605 (95.5)	
Unknown	55 (3.2)	0 (0)	55 (3.3)	

Values are presented as number (%).

**Table 2.** RR between groups exposed and not exposed to *Kudoa septempunctata*

Variables	Symptom (+)	Symptom (–)	Total (n)	RR (95% CI)^1^
Case definition 1				
Exposure	8 (29.6)	19 (70.4)	27	71.2 (27.0, 178.6)
Non-exposure	7 (0.4)	1,674 (99.6)	1,681	1.00 (reference)
Case definition 2				
Exposure	6 (22.2)	21 (77.8)	27	124.5 (43.5, 355.0)
Non-exposure	3 (0.4)	1,678 (99.6)	1,681	1.00 (reference)

Values are presented as number (%). RR, relative risk; CI, confidence interval.

1 Using the Koopman asymptotic score method.

## Data Availability

This study utilized data sourced from studies supported by the Korea Disease Control and Prevention Agency (KDCA). These data are available upon reasonable request and approval.

## Supplementary materials

Supplementary Material 1. Pre- and post- consumption questionnaire

Supplementary Material 2. Classification of Water- and Foodborne Infectious Diseases (Notifiable Infectious Diseases)

Supplementary Material 3. Reciprocal relationship and incubation period of exposed to *Kudoa septempunctata*

Supplementary Material 4. Results of PCR and culture tests for foodborne pathogens in human stool and vomit samples from symptomatic participants
