# Supplementary figures and images for: The association between the consumption of raw Kudoa septempunctata–infected farmed Paralichthys olivaceus and gastrointestinal symptoms
Source: Epidemiol Health. 2026 Jan 19;48:e2026003. doi: 10.4178/epih.e2026003 (PMC13219975; doi:10.4178/epih.e2026003)

Supplementary Material 1 Pre- and post- consumption questionnaire


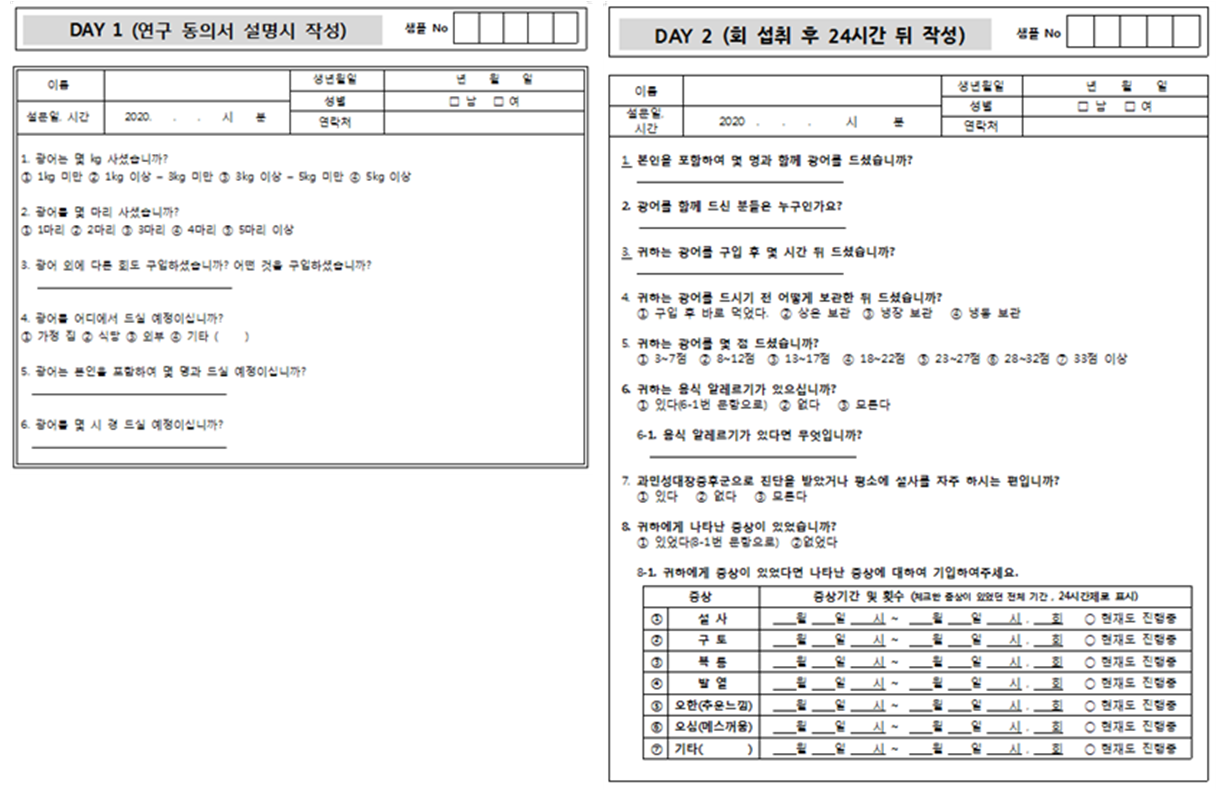

Supplement: Supplementary Material 1. — Pre- and post- consumption questionnaire [file epih-48-e2026003-Supplementary-1.docx]
