# Supplementary material for: The association between the consumption of raw Kudoa septempunctata–infected farmed Paralichthys olivaceus and gastrointestinal symptoms
Source: Epidemiol Health. 2026 Jan 19;48:e2026003. doi: 10.4178/epih.e2026003 (PMC13219975; doi:10.4178/epih.e2026003)
Supplement: Supplementary Material 2. — Classification of Water- and Foodborne Infectious Diseases (Notifiable Infectious Diseases) [file epih-48-e2026003-Supplementary-2.docx]

Supplementary Material 2 Classification of Water- and Foodborne Infectious Diseases (Notifiable Infectious Diseases)

| **구분** | | | **병원체명** |
| --- | --- | --- | --- |
| **표준**  **검사**  **항목**  **(16종)** | **세균(17종)** | **1군 감염병** | **콜레라균, 장티푸스균, 파라티푸스균, 세균성이질균, 장출혈성대장균(EAEC)** |
|  |  | **지정감염병(장관감염증)** | **살모넬라균속, 장염비브리오균, 장독소성대장균(ETEC), 장침습성대장균(EIEC), 장병원성대장균(EPEC), 캄필로박터제주니균, 클로스트리듐퍼프린젠스균, 황색포도알균, 바실루스세레우스균, 예르시니아엔테로콜리티카균, 리스테리아모노사이토제네스균** |
|  | **바이러스(6종)** | **1군 감염병** | **A형 간염바이러스^[[1]](#footnote-1)^** |
|  |  | **지정감염병(장관감염증)** | **그룹A형 로타바이러스, 아스트로바이러스, 장내아데노바이러스, 노로바이러스, 사포바이러스** |
|  | **원충(4종)** | **지정감염병(장관감염증)** | **이질아메바, 람블편모충, 작은와포자충, 원포자충** |
| **기타** | | **세균** | **장흡착성대장균(EAEC)** |
|  |  | **원충** | **쿠도아충** |

1. Hepatitis A virus was excluded from the examination items of this study [↑](#footnote-ref-1)
