# Supplementary material for: The association between the consumption of raw Kudoa septempunctata–infected farmed Paralichthys olivaceus and gastrointestinal symptoms
Source: Epidemiol Health. 2026 Jan 19;48:e2026003. doi: 10.4178/epih.e2026003 (PMC13219975; doi:10.4178/epih.e2026003)
Supplement: Supplementary Material 3. — Reciprocal relationship and incubation period of exposed to Kudoa septempunctata [file epih-48-e2026003-Supplementary-3.docx]

Supplementary Material 3 Reciprocal relationship and incubation period of exposed to *Kudoa septempunctata*

| **Raw Sample number / Exposure number** | | **Intensity of infection**  **(Spore count)** | **Amount of Intake(piece)** | **Frequency of Symptoms** | | | | | | | **Incubation period(hours)** |
| --- | --- | --- | --- | --- | --- | --- | --- | --- | --- | --- | --- |
|  |  |  |  | **diarrhea** | **vomit** | **stomach** | **fever** | **chill** | **nausea** | **other** |  |
| **A** |  | 7.5*10^^-1^/g |  |  |  |  |  |  |  |  |  |
|  | **A -1** |  | 15 | - | - | - | - | - | - | - | asymptomatic |
|  | **A -2** |  | 15 | - | - | - | - | - | - | - | asymptomatic |
| B |  | 7.5*10^-1/g |  |  |  |  |  |  |  |  |  |
|  | **B -1** |  | 20 | - | - | - | - | - | - | - | asymptomatic |
| **C** |  | 1.0x10^^0^/g |  |  |  |  |  |  |  |  |  |
|  | **C -1** |  | 10 | - | - | - | - | - | - | - | asymptomatic |
|  | **C -2** |  | 20 | - | - | - | - | - | - | - | asymptomatic |
|  | **C -3** |  | 25 | - | - | - | - | - | - | - | asymptomatic |
| **D** |  | 2.5x10^^0^/g |  |  |  |  |  |  |  |  |  |
|  | **D -1** |  | 5 | - | - | - | - | - | - | - | asymptomatic |
|  | **D -2** |  | 5 | - | - | - | - | - | - | - | asymptomatic |
|  | **D -3** |  | 15 | - | - | - | - | - | - | - | asymptomatic |
|  | **D -4** |  | 25 | - | - | - | - | - | - | - | asymptomatic |
| **E** |  | 2.5x10^^0^/g |  |  |  |  |  |  |  |  |  |
|  | **E -1** |  | 5 | - | - | - | - | - | - | - | asymptomatic |
|  | **E -2** |  | 10 | - | - | - | - | - | - | - | asymptomatic |
|  | **E -3** |  | 10 | - | - | - | - | - | - | - | asymptomatic |
| **F** |  | 5x10^^3^/g |  |  |  |  |  |  |  |  |  |
|  | **F -1** |  | 10 | - | - | - | - | - | - | - | asymptomatic |
| **G** |  | 2.8x10^^4^/g |  |  |  |  |  |  |  |  |  |
|  | **G -1** |  | 5 | - | - | - | - | - | - | - | asymptomatic |
|  | **G -2** |  | 5 | - | - | - | - | - | - | - | asymptomatic |
|  | **G -3** |  | 10 | - | - | - | - | - | - | - | asymptomatic |
| **H** |  | 5*10^^5^/g |  |  |  |  |  |  |  |  |  |
|  | **H -1** |  | 25 | - | - | - | - | - | - | - | asymptomatic |
|  | **H -2** |  | 25 | - | - | - | - | - | - | - | asymptomatic |
| **I** |  | 6.9x10^^5^/g |  |  |  |  |  |  |  |  |  |
|  | **I -1** |  | 10 |  |  |  |  |  | 1* |  | 10 hr |
|  | **I -2** |  | 15 |  |  | 1* |  |  |  |  | 10 hr |
|  | **I -3** |  | 15 |  | 10 |  | 1* | 1* | 1* |  | 4 hr |
|  | **I -4** |  | 20 |  | 3 |  | 1* | 1* | 1* |  | 4 hr |
|  | **I -5** |  | 20 | 3 | 3 |  |  |  |  |  | 4 hr |
| **J** |  | 2.5x10^^6^/g |  |  |  |  |  |  |  |  |  |
|  | **J -1** |  | 5 | 3 |  | 1* |  |  |  |  | 14 hr |
|  | **J -2** |  | 15 | 4 | 3 | 1* |  | 1* | 1* |  | 3.5 hr |
|  | **J -3** |  | 30 | 5 | 10 | 1* |  | 1* | 1* |  | 4.5 hr |
| *1 : Subjectively reported symptoms during the survey | | | | | | | | | | | |
