# Supplementary material for: The association between the consumption of raw Kudoa septempunctata–infected farmed Paralichthys olivaceus and gastrointestinal symptoms
Source: Epidemiol Health. 2026 Jan 19;48:e2026003. doi: 10.4178/epih.e2026003 (PMC13219975; doi:10.4178/epih.e2026003)
Supplement: Supplementary Material 4. — Results of PCR and culture tests for foodborne pathogens in human stool and vomit samples from symptomatic participants [file epih-48-e2026003-Supplementary-4.docx]

Supplementary Material 4 Results of PCR and culture tests for foodborne pathogens in human stool and vomit samples from symptomatic participants

| Date | Sample No. | Sample type | Bacterium | Virus | Protozoa | Result |
| --- | --- | --- | --- | --- | --- | --- |
|  |  |  | Salmonella spp., Sigella spp.,  V. parahaemolyticus, V. cholera, V. vulnificus,  EIEC, EHEC, ETEC, EPEC, EAEC, C.jejuni, C. coli,  C. perfrigens, Sta. sureus, B. cereus, L. mono cytogenes, Y. entero colitica | Noro virus,  Rota virus, Adeno virus, Astro virus,  Sapo virus | Kudoa  septempunctata |  |
| 7/6 | A-1 | Feces | Not detected | Not detected | Detection | *Kudoa septempunctata* |
| 7/6 | A -2 | Feces | Not detected | Not detected | Detection | *Kudoa septempunctata* |
| 7/6 | A -3 | Feces | Not detected | Not detected | Detection | *Kudoa septempunctata* |
| 7/8 | B-1 | Feces | Not detected | Not detected | Not detected | Not detected |
| 11/23 | C-1 | Feces | Not detected | Not detected | Not detected | Not detected |
